# On the first evidence of exchange-bias feature in magnetically contrasted consolidates made from CoFe_2_O_4_-CoO core-shell nanoparticles

**DOI:** 10.1038/s41598-019-55649-y

**Published:** 2019-12-19

**Authors:** Nancy Flores-Martinez, Giulia Franceschin, Thomas Gaudisson, Sonia Haj-Khlifa, Sarra Gam Derouich, Nader Yaacoub, Jean-Marc Grenèche, Nicolas Menguy, Raul Valenzuela, Souad Ammar

**Affiliations:** 1 Université de Paris, CNRS UMR-7086, Interfaces Traitement Organisation et DYnamique des Systèmes (ITODYS), 75013 Paris, France; 2 Le Mans Université, CNRS UMR-6283, Institut des Molécules et des Matériaux du Mans (IMMM), 72085 Le Mans, France; 3 Sorbonne Université, UMR CNRS 7590, MNHN, IRD, Institut de Minéralogie, de Physique des Matériaux et de Cosmochimie (IMPMC), 75005 Paris, France; 4 Universidad Nacional Autónoma de Mexico, Instituto de Investigaciones en materiales (IIM), 04510 Mexico City, Mexico

**Keywords:** Magnetic properties and materials, Magnetic properties and materials

## Abstract

Hetero-nanostructures based on magnetic contrast oxides have been prepared as highly dense nanoconsolidates. Cobalt ferrite-cobalt oxide core-shell type nanoparticles (NPs) were synthesized by seed mediated growth in polyol and subsequently consolidated by Spark Plasma Sintering (SPS) at 500 °C for a few minutes while applying a uniaxial pressure of 100 MPa. It is interesting to note that the exchange bias feature observed in the core-shell NPs is reproduced in their ceramic counterparts, or even attenuated. A systematic structural characterization was then carried out to elucidate the decrease in the exchange magnetic field, involving mainly advanced X-ray diffraction, zero-field and in-field ^57^Fe Mössbauer spectrometry, magnetic measurements and electron microscopy.

## Introduction

The combination of a ferro-, ferrimagnetic material (F) with an antiferromagnetic (AF) material can lead to a spin exchange coupling known as “exchange bias” EB. This phenomenon is usually characterized by an asymmetric hysteresis loop and an enhanced coercive field^1^. To be effective, this phenomenon requires nanostructures with maximum interface contact. For this reason, it is mainly focused on thin films^2–6^ and nanoparticles^7–10^ where the interface between the F and AF phases is easier to control and to characterize. Therefore, it is particularly active in the technological fields involving multilayers or embedded nanoparticles (NPs) in thin films, such as those of magnetic recording heads^11,12^, magnetoresistive random access memories (MRRAM)^13–16^, magnetic sensors^17–19^ and high storage capacity magnetic recording media^20^. There are also real potential applications for free NPs when they are in the shape of therapeutic fluids of magnetic hyperthermia or self-pumping magnetic cooling fluids or as supported granular data storage media media among others of course^21–24^.

EB is hardly considered for applications involving bulk solids like actuators or magnetic motors^25^. This lack of studies on exchange-biased consolidates is undoubtedly due to the fact that they are mainly produced by sintering but that sintering involves heating at high temperature for a long time, inducing grain growth, coarsening and hazardous atom’s diffusion, with a weak control of the interfacial crystallographic quality. As a consequence, these materials have been considered for a long time unsuitable candidates for the design of exchange-biased solids and their monolithic integration into real machines.

The emergence of Spark Plasma Sintering (SPS) technique has propelled a renewed interest on nanostructured consolidates including hetero-nanostructured consolidates, paving the way for the production of composite solids exhibiting potentially EB feature. Indeed, SPS operates at moderate temperatures for short periods of time, favoring ultrafine grained and highly dense microstructures^26,27^. It is also a powerful solid-state chemical route for the preparation of single^28–31^ and multi-phases^32–34^. The SPS technique could really overcome the limits highlighted above.

In this context, as a pioneering work, we have combined soft chemistry, particularly the polyol process, and SPS, starting from exchange-biased core-shell NPs^35^ and optimized the sintering conditions to promote large interfaces, high crystalline quality and limited atomic interdiffusion between selected F and AF phases within a single composite nanoconsolidate.

As a case study, we focused on cobalt ferrite F CoFe_2_O_4_ interfaced with cobalt oxide AF CoO, which exhibit interesting magnetic properties for the design of our desired solid and its characterization under relatively easy to achieve operating conditions. Their respective ordering temperatures (800 versus 290 K) as their magnetocrystalline anisotropy constant (1.2 versus 3.0 MJ.m^−3^) are significantly different, allowing an easy experimental evidence of EB below room temperature (r.t.)^35^.

So we present here our main structural, microstructural and magnetic characterization results, within a continuous feedback between the former properties and the latter, comparing two pristine CoFe_2_O_4_ ceramic systems and its composite counterpart CoFe_2_O_4_-CoO. For simplicity, in the further sections, cobalt ferrite and cobalt oxide phases are abbreviated as CFO and CO, respectively, while their composite is named CFO-CO.

## Results

### Structural and microstructural analysis

**Figure 1:**
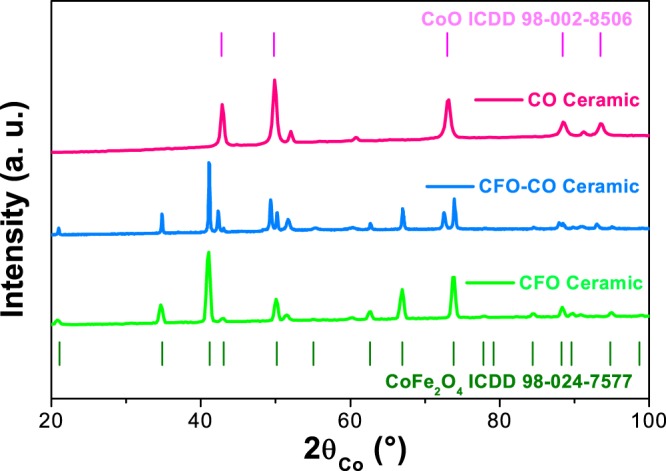
XRD pattern of CFO-CO ceramic compared to those of CFO and CO ones. The strips show the X-ray diffraction for the CoO and CoFe_2_O_4_ reference pattern 98-002-8506 and 98-024-7577, respectively. The additional peaks match with Co metal phases.

The X-ray diffraction (XRD) pattern of CFO-CO ceramic revealed four crystallographic signatures: the starting spinel cobalt ferrite and rock-salt cobalt monoxide phases, and two cobalt metal phases formed *in situ*, the face centered cubic (fcc) and the hexagonal compact (hcp) ones. This *in situ* precipitation of Co metal was also pointed out by the XRD patterns recorded on pristine CFO and CO ceramics. This demixing is assumed to be due to the reducing behavior of the SPS sintering conditions, as already highlighted in previous studies^26,27^. Interestingly, the cobalt phase structure appears to be different from one reference sample to another. Demixing cobalt from the spinel phase leads to the cubic allotrope while its demixing from the rock-salt phase leads to the hexagonal one (Fig. 1). The cobalt phase content was found to be very weak (Table 1) and comparable between that measured on the composite system and that measured on the reference ceramics. Moreover, the refined cell parameters of both the fcc and hcp Co phases were found to be very similar to those tabulated for bulk metals (ICDD n°98-062-2435 and n°98-007-6942, respectively).

**Table 1:** The cell parameter *a*, the average coherent diffraction domain length, <L_XRD_ > and the weight content of each phase in the all analyzed ceramics, as inferred from MAUD refinements^49^ (see Figure SI-1 in the supporting information section).

	CoFe_2_O_4_			CoO			fcc-Co			hcp-Co		
	*a* (Å) ± 0.002	<*L*_*XRD*_ > (nm)	Wt.-%	*a* (Å) ± 0.002	<*L*_*XRD*_>nm	Wt.-%	*a* (Å) ± 0.002	<*L*_*XRD*_ > (nm)	Wt.-%	*a* (Å) ± 0.002	<*L*_*XRD*_ > (nm)	Wt.-%
CFO	8.389	37	89	—	—	—	3.550	100^(*)^	11	—	—	—
CFO-CO	8.388	27	58	4.260	32	32	3.550	100^(*)^	7	2.500	100^(*)^	3
										4.050		
CO	—	—	—	4.260	37	95	—	—	—	2.500	100^(*)^	5
										4.050		

^(*)^ These values were fixed during MAUD refinements^49^, due to the very large *c*rystal size (more than 100 nm) of the related phase.

In addition, the refined cell parameter of CoO phase, which is about 4.260 Å for CO and CFO-CO ceramics, is very close to the tabulated value for bulk rock-salt cobalt monoxide (ICDD n°98-002-8506). This proximity makes it possible to completely neglect the demixing of hcp Co.

Finally, the refined cell parameter of the CoFe_2_O_4_ phase is equivalent from one sample to another, but slightly smaller (Table 1) than that of bulk ferrite (ICDD n°98-024-7577). The compensation of cobalt demixing in the spinel phase can be achieved under three scenarios: (i) generation of anion vacancies in the spinel lattice, (Eq. 2), (ii) partial reduction of ferric cations into ferrous ones accompanied by generation of oxygen vacancies (Eq. 2) and (iii) exclusive reduction of ferric cations into ferrous ones (Eq. 3):

1 $$CoF{e}_{2}{O}_{4}\to x\,Co+C{o}_{1-x}F{e}_{2}^{III}{O}_{4-x}$$

2 $$CoF{e}_{2}{O}_{4}\to x\,Co+C{o}_{1-x}F{e}_{2-x}^{III}F{e}_{x}^{II}{O}_{4-3x/2}$$

3 $$CoF{e}_{2}{O}_{4}\to x\,Co+C{o}_{1-x}F{e}_{2-\frac{x}{3}}^{III}F{e}_{\frac{3x}{2}}^{II}{O}_{4}$$

It is important to be able to discriminate between these three hypotheses because they can have a drastic impact on the magnetic properties of the final ceramics. Indeed, by assuming a pure inverse structure for the spinel phase, a change of its composition results in a change of its total magnetization and its magnetocrystalline anisotropy. The substitution of Co^2+^ by Fe^2+^ on octahedral spinel sites leads to an increase of magnetization, as the magnetic moment of Co^2+^ (3.3 µ_B_) is lower than that of Fe^2+^ (4.2 µ_B_). Reversely, it induces a decrease in the magnetocrystalline anisotropy constant, since the strength of the spin-orbit coupling of the coordinated octahedral Co^2+^ cation is higher than that of the coordinated octahedral Fe^2+^ cations. Now, the compensation of Co^2+^ departure by anion vacancies only leads to a decrease in both properties. The vacancies break the magnetic pathway between the paramagnetic cations. The intermediate situation, associated to the replacement of Co^2+^ by both ferrous cations and anion vacancies, leads to an intermediate magnetic response: the ferrous species reduces the strength of the spin-orbit coupling within the octahedral spinel sublattice while increasing its total magnetic moment and oxygen vacancies breaking the magnetic pathways within and between the cationic spinel sublattices.

According to the literature, it is not easy to conclude in favor of this or that hypothesis. While some groups reported the generation of oxygen vacancies in SPS sintered oxides only at very high sintering temperatures (higher than 500 °C)^36–38^, others did not exclude it on oxide ceramics prepared at 500 °C^29^. Others reported both the generation of oxygen vacancies and the precipitation of metal carbide even at sintering temperatures as low as 500 °C^39^. For instance, Velinov *et al*. observed, by SPS sintering of Ni_1-x_Zn_x_Fe_2_O_4_ nanoparticles at 500 °C, the formation of carbide resulting from the demixing of iron cations, their reduction and then their alloying with carbon atoms diffusing from the graphite die^39^. And finally, the demixing of metallic nickel, accompanied by a partial reduction of ferric cations into ferrous ones in the spinel lattice, has also been highlighted in SPS sintered ceramics (at 500 °C) Ni_1-x_Zn_x_Fe_2_O_4_^40,41^.

By focusing on our SPS operating conditions, we therefore decided to check the ability of SPS sintering to reduce ferric cations into ferrous ones. In practice, we prepared 10 nm sized maghemite Fe^III^_2_O_3_ nanoparticles in polyol and SPS sintered them at 500 °C under a uniaxial pressure of 100 MPa (see Figure SI-2 in the supporting information section). ^57^Fe Mössbauer spectra of both powder and consolidate were recorded at 300 and 77 K, to determine whether they belonged to maghemite or magnetite. ^57^Fe Mössbauer spectrometry is an iron-sensitive analytical technique able to distinguish unambiguously the chemical composition of the obtained phase using the electronic density of ^57^Fe atoms. It is interesting to note that the ceramic produced was found to be exactly consistent with a pure magnetite while its parent powder was confirmed to be a pure maghemite (see Figure SI-3). This means that during SPS processing at temperatures as low as 500 °C, ferrous cations may be formed while oxygen atoms depart from the spinel lattice stabilizing the Fe^III^_2_Fe^II^_1_O_4_ phase starting from the Fe^III^_2_O_3_ one, and thus supporting the second hypothesis instead of the first and the third^42,43^:

4 $$3F{e}_{2}{O}_{3}\to 2F{e}_{3}{O}_{4}+1/2{O}_{2}$$

In other words, the amount of ferrous cations formed *in situ* in the spinel phase during SPS sintering is equal to the amount of demixing cobalt, which in the present case is estimated at about 8 at.-% in CFO-CO ceramics (corresponding to a fcc Co weight ratio of 7 wt.-%) and about 12 at.-% in CFO ceramics (corresponding to a fcc Co weight ratio of 11 wt.-%). So, by replacing x with these values in Eq. 2, we obtain, respectively, for our CFO-based ceramics non-stoichiometric formula Co_0.92_Fe^III^_1.92_Fe^II^_0.08_O_3.88_ and Co_0.88_Fe^III^_1.86_Fe^II^_0.12_O_3.83_, explaining in a way the slight difference between the parameter of the refined spinel cells in these ceramics and the table value.

To complete our investigations, we first analyzed the ^57^Fe Mössbauer spectra of CFO and CFO-CO ceramics recorded at 300 K (above the Néel temperature of the AF phase and below the Curie temperature of the F phase), in search of ferrous cation signatures. Surprisingly, at a first lecture, the refined values for hyperfine parameters did not meet our expectations at all. The spectra are consistent with the signature of two main contributions, a sextet (major) and a doublet (minor) with an average isomer shift < δ > of 0.40 mm.s^−1^ and an average hyperfine field (<B_hyp_ > of 49.0 T) (see Figure SI-4 in the supporting information section). The sextet is quite consistent with ferric cations involved in the ferrimagnetic spinel structure but some of them with a partial electron delocalization (explaining the inner wing of the left outer line), while the quadrupolar doublet must be assigned to paramagnetic ferrous species: they result probably from a small contamination by a paramagnetic phase non detected by XRD, such as siderite FeCO_3_ (that would be compatible with the observation of traces of carbon as often reported on SPS sintered ceramics^44,45^) and/or the replacement of the CoO phase by its solid solution Co_1-x_Fe_x_O (x close to zero) (that would be compatible with the diffusion of iron cation from spinel to adjacent rock-salt grains because an interatomic diffusion in SPS sintered nanocomposites was also reported^34^).

The spectrum recorded at 77 K appeared to be better resolved, particularly the internal wings of the different lines, although the overlap between the lines still remains (Figure SI-4). For both ceramics, the spectra can be well described by means of at least three magnetic sextets (blue, red and pink lines in Figure SI-4) and a quadrupolar doublet (green line). The blue and red sextets correspond to magnetically ordered ferric cations in a specific coordination site in the spinel lattice, i.e. an octahedral and tetrahedral site (in the figure we represent the sum) according to their respective values of isomer shift (see the mean value in Figure SI-4). The pink sextet corresponds to magnetically ordered ferrous cations coordinated exclusively in octahedra (see also isomer shift value). This last component can be assigned to the diffusion of Fe^2+^ in the antiferromagnetic cobalt monoxide grains as the Néel temperature is expected to be much higher than 77 K (let us remember T_N_ (FeO, CoO) = 140 and 290 K, respectively) and/or the stabilization of such cations within the cobalt ferrite grains. Finally, the green line doublet, which is very weak and negligible (less than 3 at.-%), is consistent with paramagnetic ferrous species very probably localized within a residual siderite contaminant.

**Figure 2:**
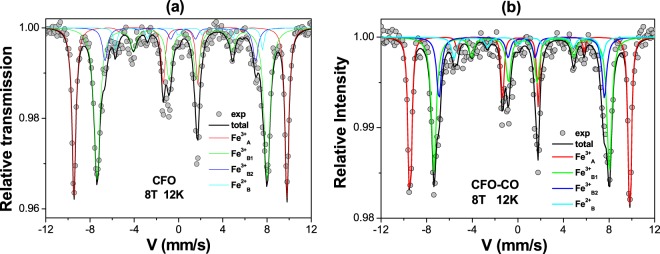
^57^Fe Mössbauer spectra collected on CFO (**a**) and CFO-CO (**b**) ceramics at 12 K, applying an 8 T magnetic field parallel to the γ-beam.

For more reliable and quantitative information, we decided to repeat our Mössbauer measurements, working under an 8 T external magnetic field (parallel to the γ beam) at a lower temperature, 12 K. The spectra obtained are unambiguously consistent with a unique ferrimagnetic structure (Fig. 2). The refinement allows attributing two magnetic sextets to the Fe^3+^ in the tetrahedral (A) and octahedral (B) sites, according to the value of isomer shift, and the third one to Fe^2+^ on B site (Table 2), in agreement with the compensation for cobalt demixing by stabilizing ferrous cations within the spinel lattice only. By applying the ferric and ferrous atomic ratios inferred from Mössbauer analysis to Eq. 2, we obtained for the spinel phase a chemical formula of (Co_0.84_Fe^III^_1.84_Fe^II^_0.16_)O_3.76_ and (Co_0.90_Fe^III^_1.90_Fe^II^_0.10_)O_3.85_ for the CFO-CO and CFO ceramics, respectively, very close to those inferred from the XRD analysis of these samples, and very close to each other, which means that both pristine and composite ceramics, exhibit more or less the same spinel phase with almost the same magnetic properties. In other words, if the former exhibits any improvement or deterioration in magnetic properties compared to the latter, it is mainly due to the presence of the AF CoO phase.

**Table 2:** Refined values of the hyperfine parameters: isomer shift (δ); quadrupole shift (2ε); effective field (B_eff_); hyperfine field (B_hyf_); average canting angle (θ) and ratio of each component evaluated from in-field Mössbauer fitted spectrum, are reported for both CFO-CO and CFO ceramics.

		T = 12 K, under external field of 8 T					
		δ (mm.s^−1^) ± 0.01	2ε (mm.s^−1^) ± 0.01	B_eff_ (T) ± 1	θ (°) ± 10	B_hyp_ (T) ± 1	Ratio (%) ± 2
CFO-CO	Fe^3+^_A_	0.37	−0.04	59.6	19	52.1	**34**
	Fe^3+^_B1_	0.55	−0.09	47.5	26	54.8	41
	Fe^3+^_B2_	0.50	0.01	44.8	0	52.8	17
	<Fe^3+^_B_>	0.54	−0.06	46.7	22	54.2	**58**
	Fe^2+^_B_	1.17	−0.20	40.0	46	35.0	**8**
CFO	Fe^3+^_A_	0.36	−0.01	59.5	11	51.7	34
	Fe^3+^_B1_	0.54	−0.08	47.4	26	54.7	53
	Fe^3+^_B2_	0.46	−0.25	42.0	17	49.7	8
	Fe^2+^_B_	1.18	−0.11	41.0	47	36.0	5

In addition, the analysis of in-field Mössbauer spectra allows an estimation of the spin canting of the involved magnetic iron species in the spinel structure. In particular, it allows the determination of the θ angle, defined by the direction of the effective field (vectorial sum of the hyperfine field and the applied field) and γ-beam direction for both A and B iron components. Typically, when the peaks of second and fifth lines do not have a zero intensity, a canted structure for iron magnetic moment with respect to the applied field exists^46,47^. On the example of CFO and CFO-CO ceramics, θ is found to be around 20–25° for Fe^3+^ cations, while it is of 46–47° for Fe^2+^ ones into the spinel lattice, meaning that the largest canting exists on the ferrous sub-lattice as a consequence on its far from thermodynamic equilibrium formation conditions.

### Magnetic analysis

The magnetic properties of CFO-CO ceramic were investigated in comparison to those of the reference CFO ceramic. The variation of the magnetization as a function of the magnetic field at different temperatures under FC and ZFC measurement conditions were recorded with a cooling magnetic field of 7 T. All the recorded isothermal M(µ_0_H) curves exhibit hysteresis loops, even at 300 K. Below the Néel temperature of the AF CoO phase, at temperatures for which EB would proceed, the coercive fields deduced from these plots are systematically higher for the composite than for the pristine ceramic.

**Figure 3:**
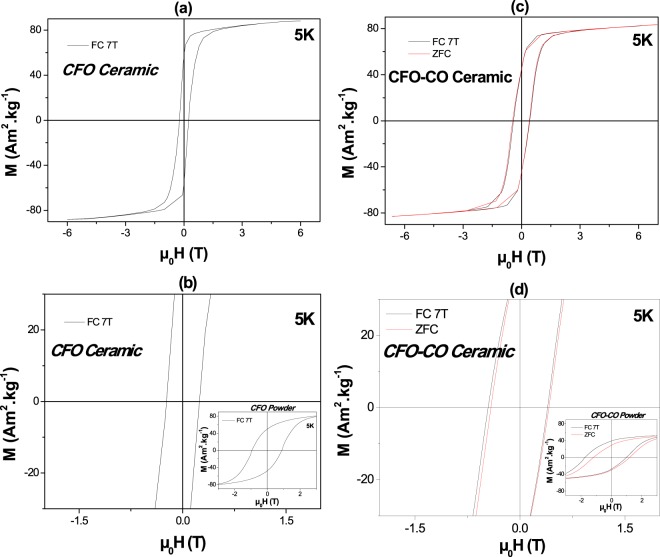
5 K M(H) hysteresis loops, and their zoom at around µ_0_H = 0, as recorded in the FC (cooling magnetic field of 7 T) and the ZFC modes on CFO (**a**,**b**) and CFO-CO (**c**,**d**) ceramics, compared in the inset to those of their nanopowder parents.

Focusing on the FC 5 K curves, well below the CoO T_N_ value (Fig. 3), both ceramics appear to correspond to the magnetic behavior of hard magnets (Table 3), but the composite one exhibits a coercive field twice than of the reference one. Also the inferred maximal magnetic energy (BH)_max_ for the composite ceramic is more than 1.5 time higher than that of the reference one (19.6 versus 12.2 kJ.m^−3^). This enhancement is associated with the evidence of EB feature with a non-zero exchange field. A µ_0_H_E_ value of 28 mT was measured on the composite ceramic, one order smaller than that previously measured, within the same operating conditions, on its CFO-CO parent powder, namely 365 mT^35^.

**Table 3:** Main magnetic characteristics of CFO-CO and CFO ceramics deduced from their hysteresis loops recorded at 5 K in the FC mode and at 300 K in the ZFC one.

	ZFC-300K			FC-5K-7T				
	M_s_ (Am^2^.kg^−1^) ± 0.5	M_r_ (Am^2^.kg^−1^) ± 0.5	µ_0_H_c_ (mT) ± 2	M_S_ (Am^2^.kg^−1^) ± 0.5	M_r_ (Am^2^.kg^−1^) ± 0.5	µ_0_H_c_ (mT) ± 2	µ_0_H_E_ (mT) ± 2	(BH)_max_ (kJ.m^−3^)
CFO	77.6	39.3	86	88.8	56.4	240	0	12.1
CFO-CO	76.0	22.0	62	83.2	48.0	430	28	19.3

It is assumed that this difference is due to the evolution of microstructural properties between powder and ceramics, and in particular to the grain size increase. Indeed, while the former exhibited a core-shell structure with a 10 nm core in size F core and a less than 2 nm in thickness AF shell (see the experimental section), with large F/AF interphase areas, the latter exhibits a kind of coagulated microstructure (see the experimental section), with more than 50 nm in size F grains surrounded by randomly arranged AF grains less than 30 nm, with significantly smaller F/AF interfaces.

It is also not excluded that in the composite ceramic, the EB coupling may also result from surface spin interactions between the F Co metal grains formed *in situ* (abbreviated as C) and those of AF CoO. But since we are certain that the size of metallic grains is much larger than that of the oxides, which means that the resulting F/AF interphase areas are significantly smaller than those developed between cobalt ferrite and cobalt oxide grains, we may assume that the C/CO coupling is negligible as compared to the CFO/CO one. Indeed, the XRD analysis allowed us to estimate the average crystal size of each constituting crystalline phase. It clearly evidenced that sizes of a few tens of nanometers were obtained for the involved spinel and rock-salt phases while sizes of some submicrometers were measured for the metal phases formed *in situ*. Transmission electron microscopy (TEM) observations performed on thinned ceramic pieces prepared by Focus Ion Beam (FIB) treatment^48^, give the same information. They showed a CFO-CO matrix with nanometer grain-size decorated with submicrometer size Co metallic precipitates (see Figure SI-5 in the supporting information section). The structural details of these domains, inferred from high resolution TEM micrographs, confirmed their fcc structure with hcp stacking faults (Figure SI-6). This must be pointed out because cubic Co metal is considered as a soft magnet. So its presence in the resulting ceramics may not induce any hysteresis loop broadening.

**Figure 4:**
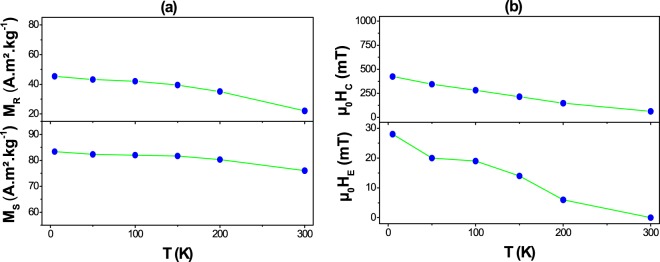
Thermal variation of the saturation and remanent magnetizations (**a**) and that of the coercive and exchange magnetic fields (**b**) of CFO-CO ceramic, as inferred from its FC hysteresis loops (cooling field of 7 T) recorded at different temperatures.

The measurement of the magnetic properties of our composite ceramics as a function of temperature showed a progressive decrease in the FC coercive and exchange fields, and consequently in the (BH)_max_, as the temperature approaches the Néel temperature of the AF CoO phase (Fig. 4 and Figure SI-7) as expected in conventional exchange-biased systems. In addition, the measured saturation (M_S_) and remanence (M_R_) magnetizations decrease as T increases (Fig. 4) but in a lighter way, particularly M_S_. Moreover, the measured M_S_ value is closer to that obtained on CFO ceramic, significantly higher than that expected for a magnetically diluted composite. At 5 and 300 K, M_S_ (assumed comparable with the magnetization value measured at 7 T) of CFO-CO reached 83 and 76 A.m².kg^−1^, respectively, very close to each other (Table 3), as a result of Co metal contamination. Thanks to its high own magnetization (170 A.m².kg^−1^ at 300 K for instance), this impurity contributes to increase the total magnetization of the composite ceramic.

**Figure 5:**
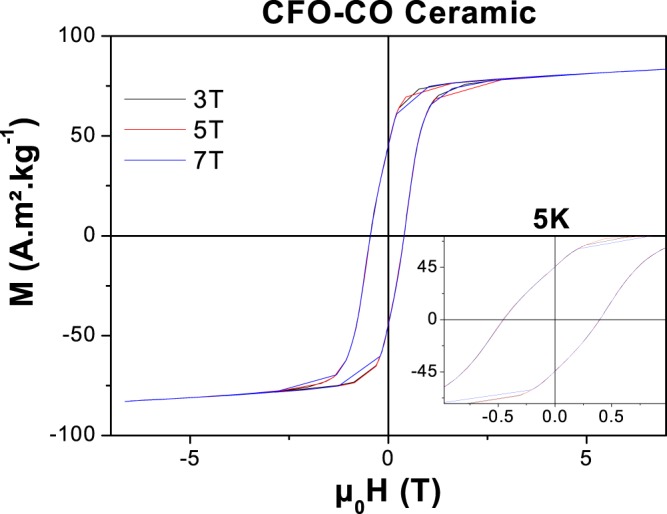
FC hysteresis loops of CFO-CO ceramic recorded at 5 K for a cooling magnetic field of 3, 5 and 7 T.

Finally, to be as exhaustive as possible in the magnetic characterization of our exchange-biased granular solid, we recorded its 5 K FC hysteresis loops, applying different cooling fields, 7, 5 and 3 T (Fig. 5). These values were chosen much higher than the coercive field previously measured at 5 K, in order to avoid minor loops features. The main magnetic characteristics deduced from these curves are summarized in Table 4. It is interesting to note that all µ_0_H_c_, µ_0_H_E_, M_R_ and M_S_ measured values are equivalent, from one curve to another, regardless of the cooling field value. Focusing on the µ_0_H_E_ values, it seems that they reach a kind of plateau in the explored magnetic cooling field range. Such saturation is consistent with EB, definitely excluding any other physical phenomena such as the effect of minor loops, the glass effect or others as a possible origin of the observed FC hysteresis loop shift on our magnetically contrasted nanoconsolidate.

**Table 4:** Dependence of the main 5 K magnetic characteristics of CFO-CO ceramic on the applied cooling magnetic field.

	FC-5K					
	µ_0_H_cooling_ (T)	µ_0_Hc (mT) ± 2	µ_0_H_E_ (mT) ± 2	M_R_ (A.m².kg^−1^) ± 0.5	M_S_ (A.m².kg^−1^) ± 0.5	(BH)_max_ (kJ.m^−3^)
CFO-CO	3	429	30	45.5	80.7	19.3
	5	424	30	45.4	81.0	19.3
	7	424	28	45.4	83.8	19.3

## Conclusions

Starting from exchange-biased CoFe_2_O_4_-CoO core-shell nanoparticles prepared by polyol route, we successfully prepared their nanoconsolidate composite by SPS. During sintering, both the microstructure and the chemical composition of the starting powder changed and a highly dense (>90%) and ultrafine grained (<100 nm) ceramics with a nanoaggregate morphology were obtained. Also, due to the reducing condition of SPS, demixing of cobalt from the spinel phase proceeded leading to the precipitation of a small amount (around 15 at.-%) of about 500 nm sized Co metal. As a consequence, the initial stoichiometric phase CoFe_2_O_4_ changed into a non-stoichiometric Co_0.84_Fe^III^_1.84_Fe^II^_0.16_O_3.76_. The presence of highly magnetized Co metal possessing a high magnetization value affects the total composite magnetization, since it compensates for the decrease of magnetization due to the AF CoO grains. It does not affect significantly its magnetic anisotropy. Its large size, compared to that of CoO grains, makes the EB coupling between its spin surface and that of CoO not significant. Only the interfaces shared between CFO and CO grains cause EB onset at low temperature, with an exchange field value, µ_0_H_E_, of about 30 mT at 5 K, regardless of the value of cooling magnetic field (3, 5 or 7 T). This EB value is lower than that measured on the starting composite powder, because of the crystal size increase in the F and AF phases as well as the evolution of the chemical composition of the spinel phase.

## Materials and Methods

### Chemicals

Fe(CH_3_CO_2_)_2_, Co(CH_3_CO_2_)_2_.4H_2_O metal salts precursors and HO(CH_2_)_2_O(CH_2_)_2_OH (DEG, b.p. = 245 °C), HO(CH_2_)_2_O(CH_2_)_2_O(CH_2_)OH (tEG, b.p. = 285 °C) solvents were purchased from ACROS and used without purification.

### Ceramics fabrication

**Figure 6:**
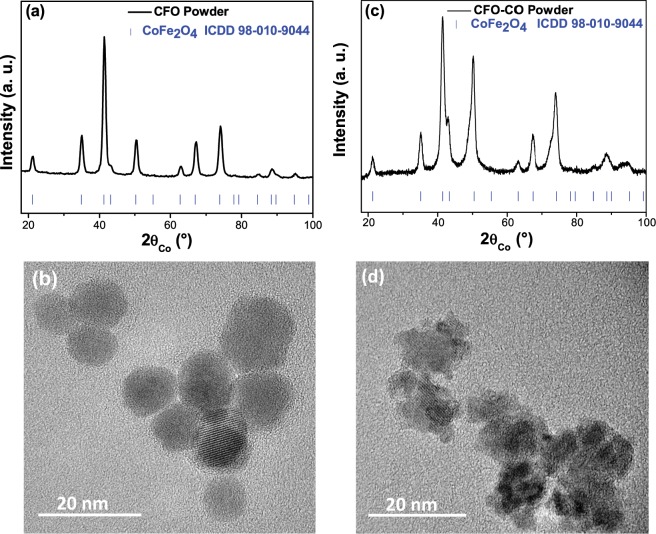
XRD patterns of CFO (**a**) and CFO-CO (**c**) particles and their respective representative high resolution transmission electron microscopy images (**b**,**d** respectively).

CFO, CO and CFO-CO ceramics were prepared by SPS starting from their polyol-made nanoparticle parents. Typically, 10 nm sized CFO particles were first precipitated in polyol and used as seeds in a fresh cobalt salt polyol solution to deposit around them a thin poly-and nanocrystalline CO shell leading to about 12 nm sized CFO-CO core-shell particles, with a CFO and CO weight content of about 70 and 30 wt.-%, respectively (Fig. 6)^35^.

Then, 1 g of each CO, CFO and CFO-CO powder was introduced into a Ø 8 mm carbon die with a layer of protective graphite foil. The die was closed by carbon punches at both sides, which transmit an initial uniaxial pressure of 50 MPa. Direct current pulses were supplied to the die by the punches using DR. SINTER515S SYNTEX SPS machine (Thiais, France) allowing the temperature to reach a first plateau of 280 °C (10 min) by gradually increasing the pressure from 50 to 100 MPa and by desorbing the residual organic species^26,27^. The temperature was then increased up to 500 °C in 5 min and maintained at that temperature for 5 min. The consolidation process was followed by recording the derivative of the distance between crucible pistons, dz/dt, with time, during the heating (not shown). The appearance of a maximum in the dz/dt(t) curve means that the sintering is processing. For both CFO and CFO-CO powders, this maximum occurs between 450 and 500 °C, which is sufficiently low to avoid or at least limit grain growth.

**Figure 7:**
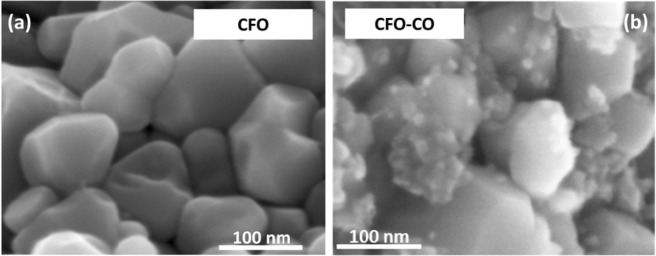
SEM micrographs of CFO (**a**) and CFO-CO (**b**) ceramics showing typically well-welded polygonal shaped grains.

The consolidation success of these powders was confirmed by Scanning Electron Microscopy (SEM) thanks to a Supra40 ZEISS FEG-SEM microscope operating at 5.0 kV. A typical highly dense (>90%) and ultrafine grained (<100 nm) microstructure was systematically evidenced in agreement with our expectations (Fig. 7). In addition, in the case of the CFO-CO ceramic produced, typical characteristics of a nanocomposite were observed: their micrographs consisted of a combination of a coagulated morphology with a polygonal morphology.

### Structural and microstructural characterizations

The crystalline structure of all the prepared ceramics was checked by X-ray Diffraction (XRD) using a Panalytical X’pert Pro diffractometer, working in the Bragg-Brentano θ-θ reflexion geometry, and equipped with a multichannel X’celerator detector and a cobalt X-ray tube operating at 40 kV and 40 mA. The collected data were then analyzed by the Rietveld method using MAUD software version 2.92 (http://maud.radiographema.eu/). The fitting was completed when the starting structural model converged satisfactorily with a R_B_ reliability factor and a χ^2^ coefficient very close to 2% and 1, respectively. To determine more precisely the structure of the involved phases, ^57^Fe Mössbauer spectrometry was carried out at 300 and 77 K in a zero magnetic field and at 12 K in a 8 T magnetic field oriented parallel to the γ-beam, using a ^57^Co/Rh γ-ray source, mounted on a conventional constant acceleration vibrating electromagnetic transducer. In all cases, the spectra were analyzed by least-squares fitting model using lorentzian lines, to determine for each involved iron species its characteristic isomer shift *δ* (referring to the isomer shift of standard α-Fe at 300 K), quadrupolar shift *2ε* and hyperfine field *B*_*hyp*_ parameters.

### Magnetic characterization

The magnetic properties of the produced ceramics were measured by a Quantum Design PPMS magnetometer. Their isothermal 5 K dc-magnetization *M* was measured by cycling the magnetic field µ_0_*H* between +7 and −7 T, under both zero-field-cooling (ZFC) field-cooling (FC) condition. In the last case, the samples were cooled from 400 to 5 K under an external field of 7 T. The recorded *M*(µ_0_*H*) data were then simultaneously plotted to evidence any horizontal loop shift as a signature of EB onset. The exchange field *H*_*E*_ is then determined as a function of *H*_*c+*_ and *H*_*c-*_, the positive and negative value of the coercivity:

5 $${H}_{E}=-\,1/2({H}_{c-}+{H}_{c+})$$

To be as exhaustive as possible, the same measurements were repeated at 50, 100, 150, 200 and 250 K to follow the thermal effect on *H*_*E*_ value, as well as for different cooling magnetic fields to confirm the presence of the EB feature.

## Supplementary information

Supplementary Information

## References

[1] Nogués J (2005). Exchange bias in nanostructures. Phys. Rep..

[2] Pan L, Soh WT, Phuoc NN, Ong CK (2018). Non-resonant and Resonant Spin Rectification in the Exchange Biased NiFe/MnIr Bilayer. Phys. Status Sol.: Rapid Res. Lett..

[3] O’Grady K, Fernandez-Outon LE, Vallejo-Fernandez G (2010). A new paradigm for exchange bias in poly-crystalline thin films. J. Magn. Magn. Mater..

[4] Mallick K, Kumar PSA (2018). Crystallite size dependent exchange bias in MgFe_2_O_4_ thin films on Si(100). J. Appl. Phys..

[5] Huang J, Gellatly A, Kauffmann A, Sun X, Wang H (2018). Exchange Bias Effect along Vertical Interfaces in La_0.7_Sr_0.3_MnO_3_:NiO Vertically Aligned Nanocomposite Thin Films Integrated on Silicon Substrates. Cryst. Grow. Design,.

[6] Lim S-H (2009). Exchange bias in thin-film (Co/Pt)_3_/Cr_2_O_3_ multilayers. J. Magn. Magn. Mater.,.

[7] Gaudisson T (2014). Combined TEM and XPS analyses to elucidate the fine Microstructure of exchange-biased oxide based core@shellnanoparticulesProduced by Seed Mediated Growth in Polyol. J. Nanopart. Res..

[8] Franceschin, G. *et al*. Exchange-Biased Fe_3−x_O_4_‒CoO granular composites of different morphologies prepared by seed-mediated growth in polyol: From core-shell to multicore embedded structures. *Part*. *Part*. *Syst*. *Charact*. 1800104 (2018).

[9] Lavorato GC, Lima E, Troiani HE, Zysler RD, Winkler EL (2017). Tuning the coercivity and exchange bias by controlling the interface coupling in bimagnetic core/shell nanoparticles. Nanoscale.

[10] Mohan R, Ghosh MP, Mukherjee S (2018). Large exchange bias effect in NiFe_2_O_4_/CoO nanocomposites. Mater. Res. Express.

[11] Baibich MN (1988). Giant magnetoresistance of (001)Fe/(001)Cr magnetic superlattices. Phys. Rev. Lett..

[12] Dieny B (1991). Giant magnetoresistive in soft ferromagnetic multilayers. Phys. Rev. B.

[13] Parkin SSP (1999). J. Appl. Phys..

[14] Wu SM (2010). Reversible electric control of exchange bias in a multiferroic field-effect device. Nat. Mater..

[15] Yuasa S, Hono K, Hu G, Worledge DC (2018). Materials for spin-transfer-torque magnetoresistive random-access memory. MRS Bull..

[16] Levartoski de Araujo C, Alves S, Buda-Prejbeanu L, Dieny B (2016). Multilevel Thermally Assisted Magnetoresistive Random-Access Memory based on exchange-biased vortex cConfigurations. Phys. Rev. Appl..

[17] Cheng Y, Peng B, Hu Z, Zhou Z, Liu M (2018). Recent development and status of magnetoelectric materials and devices. Phys. Lett. A.

[18] Apicella V, Caponero MA, Davino D, Visone C (2018). A magnetostrictive biased magnetic field sensor with geometrically controlled full-scale range. Sensors & Actuators A.

[19] Dohmeier N (2018). Inverse magnetostrictive stress sensors based on crossed pinned CoFeB/MgO/CoFeB tunnel junctions. J. Appl. Phys.,.

[20] Skumryev V (2003). Beating the superparamagnetic limit with exchange bias. Nature.

[21] Khurshid H (2015). Anisotropy effects in magnetic hyperthermia: A comparison between spherical and cubic exchange-coupled FeO/Fe_3_O_4_ nanoparticles. J. Appl. Phys..

[22] Joseph A, Mathew S (2014). Ferrofluids: Synthetic strategies, stabilization, physicochemical features, characterization and applications. ChemPlusChem..

[23] Lisjak D, Mertelj A (2018). Anisotropic magnetic nanoparticles: A review of their properties, syntheses and potential applications. Prog. Mater. Sci..

[24] Wu L, Mendoza-Garcia A, Li Q, Sun S (2016). Organic phase syntheses of magnetic nanoparticles and their applications. Chem. Rev..

[25] Luborsky FE (1966). Permanent Magnets in Use Today. J. Appl. Phys..

[26] Gaudisson T (2014). On the microstructural and magnetic properties of fine-grained CoFe_2_O_4_ ceramics produced by combining polyol process and spark plasma sintering. J. Magn. Magn. Mater..

[27] Gaudisson T (2015). Ultrafine grained high density manganese zinc ferrite produced using polyol process assisted by Spark Plasma Sintering. J. Magn. Magn. Mater.,.

[28] Gaudisson T (2013). Combining soft chemistry and Spark Plasma Sintering to produce highly dense and finely grained soft ferrimagnetic Y_3_Fe_5_O_12_ (YIG) Ceramics. J. Am. Ceram. Soc..

[29] Regaieg Y (2014). Magnetic and magnetocaloric properties of La_0.85_(Na_1−x_K_x_)_0.15_MnO_3_ ceramics produced by reactive spark plasma sintering. J. Appl. Phys.,.

[30] Ayadi F (2018). Importance of the synthesis and sintering methods on the properties of manganite ceramics: the example of La_0.7_Ca_0.3_MnO_3_. J. All. Compds..

[31] Vázquez-Victorio G (2018). Low-temperature short-time SPS processes to produce fine-grained high-coercivity Barium hexaferrite ceramics from polyol-made nanoparticles. J. Superconductivity Novel Magn.,.

[32] Gaudisson T, Ammar S, LoBue M, Mazaleyrat F (2013). Giant Barkhausen jumps in exchange-biased bulk nanocomposites sintered from core-shell Fe_3_O_4_−CoO nanoparticles. IEEE Trans. Magn.,.

[33] Acevedo Salas U (2017). An impedance spectroscopy study of magnetodielectric coupling in BaTiO_3_-CoFe_2_O_4_ nanostructured multiferroics. AIP Adv..

[34] Franceschin G (2019). On the limits of Reactive-Spark-Plasma Sintering to prepare magnetically enhanced nanostructured ceramics: the case of the CoFe_2_O_4_-NiO system. Sci. Rep..

[35] N. Flores-Martinez, *et al*, Giant Exchange-Bias in Polyol-Made CoFe_2_O_4_-CoO Core–Shell Like Nanoparticles. *Part*. *Part*. *Syst*. *Charact*., 1800290 (2018).

[36] Acevedo-Salas U (2017). Nanostructured tetragonal barium titanate produced by the polyol and spark plasma sintering (SPS) route. Appl. Phys. A.

[37] Beltrán H, Prades M, Masó N, Cordoncillo E, West AR (2010). Voltage-Dependent Low-Field Bulk Resistivity in BaTiO_3_:Zn Ceramics. J. Am. Ceram. Soc..

[38] Zhang H, Kim B-N, Morita K, Keijiro Hiraga HY, Sakka Y (2011). Effect of sintering temperature on optical properties and microstructure of translucent zirconia prepared by high-pressure spark plasma sintering. Sci. Technol. Adv. Mater.,.

[39] Velinov N (2012). Spark plasma sintering synthesis of Ni_1−x_Zn_x_Fe_2_O_4_ ferrites: Mössbauer and catalytic study. Solid State Sci..

[40] Valenzuela R, Beji Z, Herbst F, Ammar S (2011). Ferromagnetic resonance investigation of SPS-Sintered Ni-Zn nanoparticles produced by a chemical route. J. Appl. Phys..

[41] Valenzuela R, Gaudisson T, Ammar S (2016). Severe reduction of Ni-Zn ferrites during consolidation by Spark Plasma Sintering (SPS). J. Magn. Magn. Mater..

[42] Righi G, Magri R (2019). Reduction and oxidation of maghemite (001) Surfaces: the Role of Iron Vacancies. J. Phys. Chem. C.

[43] Gotic M, Košcec G, Musi S (2009). Study of the reduction and reoxidation of substoichiometric magnetite. J. Mol. Struct..

[44] Morit K, Kim B-N, Yoshida H, Hiraga K, Sakka Y (2015). Assessment of carbon contamination in MgAl_2_O_4_ spinel during spark-plasma-sintering (SPS) processing. J. Ceram. Soc. Jap..

[45] Morit K, Kim B-N, Yoshida H, Hiraga K, Sakka Y (2018). Distribution of carbon contamination in oxide ceramics occurring during spark-plasma-sintering (SPS) processing: Effect of SPS and loading temperatures. J. Eur. Ceram. Soc..

[46] Coey JMD (1971). Non-collinear spin arrangement in ultrafine ferrimagnetic crystallites. Phys. Rev. Lett..

[47] Tronc E, Prené P, Jolivet J-P, Dormann J-L, Greneche J-M (1997). Spin canting in γ-Fe_2_O_3_. Hyperfine Interact..

[48] Huguet-Garcia J (2015). Study of the Ion Irradiation Behavior of Advanced SiC Fibers by Raman Spectroscopy and Transmission Electron Microscopy. J. Am. Ceram. Soc..

[49] Lutterotti L, Matthies S, Wenk HR (1999). MAUD: a friendly Java program for material analysis using diffraction. IUCr CPD Newslett..

