# Supplementary material for: On the first evidence of exchange-bias feature in magnetically contrasted consolidates made from CoFe2O4-CoO core-shell nanoparticles
Source: Sci Rep. 2019 Dec 19;9:19468. doi: 10.1038/s41598-019-55649-y (PMC6923415; doi:10.1038/s41598-019-55649-y)
Supplement: Supplementary file 1 — Supplementary Information [file 41598_2019_55649_MOESM1_ESM.docx]

On the first evidence of exchange-bias feature in magnetically contrasted consolidates made from CoFe_2_O_4_-CoO core-shell nanoparticles

**Nancy Flores-Martinez^1*^, Giulia Franceschin^1^, Thomas Gaudisson^1^, Sonia Haj-Khlifa^1^, Sarra Gam Derouich^1^, Nader Yaacoub^2^, Jean-Marc Grenèche^2^, Nicolas Menguy^3^, Raul Valenzuela^4^, Souad Ammar ^1^**^*^

^1^ Université Paris Diderot, Sorbonne Paris Université, CNRS UMR-7086, Interfaces Traitement Organisation et DYnamique des Systèmes (TODYS), 75013 Paris, France.

^2^ Université du Mans, CNRS UMR-6283, Institut des Molécules et des Matériaux du Mans (IMMM), 72085 Le Mans, France.

^3^ Sorbonne Université, UMR CNRS 7590, MNHN, IRD, Institut de Minéralogie, de Physique des Matériaux et de Cosmochimie (IMPMC), 75005 Paris, France.

^4^ Universidad Nacional Autónoma de Mexico, Instituto de Investigaciones en materiales (IIM), 04510 Mexico City, Mexico.

* [flornatur@gmail.com](mailto:flornatu@gmail.com), ammarmer@univ-paris-diderot.fr

**Supporting information**

Figure SI-1. Experimental (scatter) and calculated (red line) XRD patterns of CFO (a) and CFO-CO (b) ceramics. The residue, defined as the difference between the experimental and calculated diffractograms, is given for each sample (blue line) to illustrate the fit quality. The reached Bragg reliability factor R_B_ ranges between 1 and 2 for all the performed refinements. Note, a pseudo-Voigt function was used for modelling the peak profile. A set of about 20 parameters was then refined, including the scale factor, the zero shift, the background polynomial coefficients, the peak profile parameters, and for each constituting phase, the cell parameter, the average crystallographic coherence length, assuming isotropic crystals, the average lattice micro-deformation, the weight ratio and the atomic isotropic temperature factors. The atomic coordinates of all the atoms except O in the spinel phase have been fixed. In order to converge rapidly the fitting process, the occupation site ratios were firstly set in relation to the nominal chemical composition for each phase. The close proximity of the atomic number between iron and cobalt atoms does not allow an accurate estimation of their distribution along the considered crystallographic lattices.

Figure SI-2. a) XRD pattern of the magnetite ceramic (FO) matching very well with the spinel structure and b) its SEM micrograph.

Figure SI-3. ^57^Mössbauer spectra of a) maghemite powder recorded at 77 K and b) magnetite ceramic recorded at 300 K. The spectra were analysed by least-squares fitting model using lorentzian lines, to determine for each involved iron species its characteristic hyperfine parameters (see Table SI-1).

|  | Maghemite nanopowder | | | |  | Magnetite nanoceramic | | | |
| --- | --- | --- | --- | --- | --- | --- | --- | --- | --- |
|  | 77K | | | |  | 300K | | | |
|  | δ  (mm.s^-1^) | 2ε (mm.s^-1^) | B_hyp_ (T) | Atomic ratio (%) |  | δ  (mm.s^-1^) | 2ε (mm.s^-1^) | B_hyp_ (T) | Atomic ratio (%) |
| Fe^3+^_B_ (red) | 0.45 | 0.00 | 51.7 | 64 | Fe^2+^ (red) | 0.36 | 0.02 | 48.9 | 34 |
| Fe^3+^_A_ (blue) | 0.41 | -0.02 | 49.4 | 36 | Fe^3+^ (blue) | 0.45 | 0.06 | 45.6 | 66 |

**Table SI-1.** Main refined Mösbauer parameters of both maghemite powder and magnetite ceramics : isomer shift *δ* (referring to the isomer shift of standard α-Fe at 300 K), quadrupolar shift *2ε* and hyperfine field *B_hyp_* parameters and its atomic ratio.

**Figure SI-4.** Mössbauer spectra recorded at 300 K (bottom) and 77 K (up) on CFO (a) and CFO-CO (b) ceramics. The scatters and the continuous black line correspond to the experimental data and the total calculated ones, respectively, assuming different iron contributions. Typically, at 300 K, a magnetically ordered ferric contribution (blue line) and a disordered ferrous one (green line) are needed to reproduce the experimental spectrum. At 77 K, two magnetically ordered ferric contributions (the red and blue lines for the Fe^3+^ in tetrahedrally and octahedrally coordinated cations in the spinel lattice and the pink line for the Fe^2+^ in octahedrally ones) and non-magnetically ordered ferrous one (green line). The main refined 77 K hyperfine parameter values are given in table SI-2.

|  |  | **T = 77 K without an external magnetic field** | | | |
| --- | --- | --- | --- | --- | --- |
|  |  | δ (mm.s^-1^)  ± 0.01 | 2ε (mm.s^-1^)  ± 0.01 | B_hyp_ (T)  ± 1 | Ratio (%)  ± 2 |
| **CFO-CO** | Fe^3+^ | 0.53 | -0.04 | 54.0 | 36 |
|  | Fe^3+^ | 0.38 | 0.00 | 51.0 | 56 |
|  | Fe^2+^ | 1.14 | 1.62 | 35.7 | 5 |
|  | Fe^2+^ | 1.26 | 2.34 | - | 3 |
| **CFO** | Fe^3+^ | 0.54 | -0.01 | 54.2 | 36 |
|  | Fe^3+^ | 0.41 | -0.01 | 51.3 | 60 |
|  | Fe^2+^ | 1.16 | 2.18 | 37.4 | 3 |
|  | Fe^2+^ | 1.26 | 2.78 | - | 1 |

**Table SI-2.** Main refined Mösbauer parameters of both CFO and CFO-CO eramics : isomer shift *δ* (referring to the isomer shift of standard α-Fe at 300 K), quadrupolar shift *2ε* and hyperfine field *B_hyp_* parameters and its atomic ratio.

**Figure SI-5**. STEM HAADF (a, b) and STEM-XEDS (c-f) of one CFO-CO ceramic. The very bright areas in (a), (b) and (c) correspond to metallic cobalt surrounded by a CFO matrix containing very small (≈ 30 nm sized) CoO nanoparticles. In the composite RGB image (f), the metallic cobalt, the CFO matrix and the CoO particles appear respectively to red, cyan and purple areas. A JEOL 2100F microscope operating at 200 kV and equipped with a Schottky emission gun, a high resolution UHR pole piece and a Gatan US4000 CCD camera was used to achieve these observations. It was also equipped by a XEDS JEOL detector coupled with a STEM device.

**Figure SI-6.** (a) TEM bright field images of a metallic Co^0^ grain (see chemical mapping on the inset). (b) HRTEM related to the yellow dashed square area labelled in (a) revealing numerous defects and stacking faults within the Co crystal. The selecting area electron diffraction (SAED) pattern corresponding to the area (b) exhibits well defined diffraction peaks and streaks along one direction related to stacking faults and possible twins within the Co crystal. (d) Crystallographic analysis shows that the complex SAED may be explained as the superimposition of fcc-Co <110> and hcp-Co <110> zone axis diffraction patterns and double diffraction phenomenon. (e) HRTEM of a single grain (i.e. without fault or defects), the corresponding Fast Fourier Transform (FFT) is related to a fcc-Co <110> zone axis diffraction pattern.

**Figure SI-7.** FC-hysteresis loops of CFO-CO ceramic recorded at different temperature for a cooling magnetic field of 7T. Magnification of the low-field section is given in the inset to show the detail of an eventual asymmetry in the magnetic field axis. The main magnetic parameters inferred from these curves are summarized in Table SI-3.

| T  K | µ_0_H_c_(FC,7T)  mT  ± 2 | µ_0_H_E_  mT  ± 2 | MR_r_(FC,7T)  A.m².kg^-1^  ± 0.5 | M_S_FC,7T)  A.m².kg^-1^  ± 0.5 | (BH)_max_  kJ.m^-3^ |
| --- | --- | --- | --- | --- | --- |
| 5 | 424 | 28 | 45.3 | 83.4 | 19.3 |
| 50 | 344 | 20 | 43.2 | 82.3 | 11.7 |
| 100 | 282 | 19 | 42.0 | 82.0 | 10.2 |
| 150 | 214 | 14 | 39.4 | 81.7 | 6.6 |
| 200 | 147 | 6 | 35.1 | 80.3 | - |
| 300 | 62 | 0 | 22.0 | 76.0 | 1.1 |

**Table SI-3.** µ_0_H_C_, µ_0_H_E_, M_R_, M_S_ and (BH)_max_ magnetic parameters measured at different temperatures on the CFO-CO ceramic for a cooling magnetic field of 7T.
